# New insights into the synergism of nucleoside analogs with radiotherapy

**DOI:** 10.1186/1748-717X-8-223

**Published:** 2013-09-26

**Authors:** Michael W Lee, William B Parker, Bo Xu

**Affiliations:** 1 Department of Medical Education, College of Medicine, University of Central Florida, 6850 Lake Nona Blvd., Orlando, FL 32827, USA; 2 Department of Biochemistry and Molecular Biology, Southern Research Institute, Birmingham, AL 35205, USA; 3 Comprehensive Cancer Center, University of Alabama at Birmingham, Birmingham, AL 35205, USA; 4 Laboratory of Molecular Radiation biology, Southern Research Institute, 2000 9th Ave South, Birmingham, AL 35205, USA

**Keywords:** Nucleoside analogs, Radiotherapy, Deoxycytidine kinase, ATM

## Abstract

Nucleoside analogs have been frequently used in combination with radiotherapy in the clinical setting, as it has long been understood that inhibition of DNA repair pathways is an important means by which many nucleoside analogs synergize. Recent advances in our understanding of the structure and function of deoxycytidine kinase (dCK), a critical enzyme required for the anti-tumor activity for many nucleoside analogs, have clarified the mechanistic role this kinase plays in chemo- and radio-sensitization. A heretofore unrecognized role of dCK in the DNA damage response and cell cycle machinery has helped explain the synergistic effect of these agents with radiotherapy. Since most currently employed nucleoside analogs are primarily activated by dCK, these findings lend fresh impetus to efforts focused on profiling and modulating dCK expression and activity in tumors. In this review we will briefly review the pharmacology and biochemistry of the major nucleoside analogs in clinical use that are activated by dCK. This will be followed by discussions of recent advances in our understanding of dCK activation via post-translational modifications in response to radiation and current strategies aimed at enhancing this activity in cancer cells.

## Overview of purine and pyrimidine nucleoside analogs that synergize with ionizing radiation

Nucleoside analogs comprise a class of rationally designed agents that emerged in the 1950s from insight gained by advances made in understanding DNA structure and DNA synthesis. In many ways, the underlying logic behind the creation of these compounds presaged the development of more recent targeted therapies by modeling cancer drugs after endogenous nucleotides in an effort to corrupt key cellular processes. By acting in this manner, it has become possible to kill rapidly dividing cancer cells by exploiting differences in the rate and amount of DNA synthesis between normal cells and cancer cells. The nucleoside analogs can be divided into sub-classes based on their structural similarity to purine bases (adenine and guanine) or pyrimidine bases (cytosine, uracil, or thymine). In general, these agents exert their cytotoxic actions through common means such as disruption of DNA function, inhibition of DNA replication, or a combination thereof. Additionally, these drugs as a class share the need to be transported into the cell through nucleoside transporters and are metabolically activated following internalization into the cell. A number of these drugs have been shown to work synergistically with radiation, a feature that is exploited clinically to enhance tumor regression, and the subject of this review. We will briefly review the pharmacology for each of these compounds followed by putative mechanisms by which radiosensitization or chemosensitization may be achieved. We will conclude with a discussion of recent efforts to identify patient suitability for combination chemotherapy and ways to enhance these synergistic effects in susceptible individuals in an optimal fashion.

### Purine based analogs

#### Fludarabine

Originally synthesized by John Montgomery and Kathleen Hewson in 1969, the fluorinated arabinosyl nucleoside analog fludarabine is a prodrug that incorporates a number of structural features that extend its half-life by protecting it from degradative enzymes [1]. Some of these structural features are shared by other nucleoside analogs. For example, the presence of a fluorine atom on the 2- position of the adenine ring makes fludarabine resistant to ADA mediated metabolism. The presence of a hydroxyl group (β instead of α) at the 2-position of the sugar ring is also a common structural modification that helps reduce glycosidic bond cleavage by the bacterial purine nucleoside phosphorylase (PNP), although fludarabine is still susceptible to phosphorolysis by human PNP [2]. Degradation by bacterial PNP can limit oral bioavailability whereas human PNP is involved in normal cellular metabolism of purine nucleosides to bases [3]. Unlike other nucleoside analogs, fludarabine is administered in the monophosphate form to increase solubility and bioavailability [4,5]. However, fludarabine-monophosphate is rapidly dephosphorylated by plasma localized 5′-nucleotidases in a rapid and complete fashion prior to cellular uptake by the hENT1, hENT2, hCNT2, or hCNT3 nucleoside transporters [5]. Once internalized into the target cell, fludarabine is phosphorylated by deoxycytidine kinase (dCK) to a monophosphate form facilitating its retention inside the cell. Phosphorylation of fludarabine to the diphosphate and triphosphate forms appear to be catalyzed by adenylate kinase and nucleoside diphosphate kinase, respectively [6]. The affinity of dCK for fludarabine appears to be relatively weak as evidenced by low Km values (100-600 μmol/L) and this may be explained by the aforementioned structural modifications [6-9]. Nevertheless, the interaction of dCK with fludarabine is specific and occurs rapidly when dCK is abundant [6]. As a consequence T-lymphoblasts, which contain high levels of dCK, are particularly sensitive to fludarabine due to the increased production of fludarabine-monophosphate [10]. At the present time, however, the major clinical utility of fludarabine is for the treatment of refractory chronic lymphocytic leukemia (CLL) [11]. Fludarabine has been tested for efficacy against a wide variety of solid tumors in the absence of radiation yielding unimpressive results. This may be potentially explained by the exceedingly low levels of dCK activity seen in non-lymphoid tissues [10]. However, more recent efforts using fludarabine in combination with radiation have shown promise in treating non-small cell lung cancer (NSCLC) and head and neck squamous cell carcinomas (HNSCC), at least in terms of tolerability and safety [12]. The major molecular actions of fludarabine tri-phosphate, which lead to cytotoxicity and radiosensitivity, may be explained in part by inhibition of DNA polymerases and inhibition of ribonucleotide reductase with consequent depletion of deoxynucleotide pools [6]. Incorporation of fludarabine into DNA can lead to chain termination and induction of apoptosis in a cell cycle specific manner [13]. Alternative mechanisms relating to inhibition of DNA repair machinery have been proposed to explain cell death initiation in quiescent tumor cells in response to fludarabine [14].

#### Cladribine

Cladribine, a chlorinated deoxyadenosine nucleoside analog, has been the agent of choice in the treatment of hairy cell leukemia since the early 1990s [15]. Cladribine has also demonstrated utility in the treatment of chronic myelogenous leukemias and non-hodgkins lymphomas but, similar to what has been reported for fludarabine, it has not produced impressive outcomes with solid tumors [16-18]. The synthesis of cladribine in the 1960s grew out of efforts to produce agents with enhanced cytotoxicity and decreased susceptibility to catabolism using insight gained from studies of agents like ara-A [19]. Indeed, the chloride atom placed at the 2-position of the adenine ring is a key modification that interferes with catabolism through inhibition of deamination by ADA [20]. However, oral administration of cladribine is hindered by poor oral bioavailability due to degradation by the actions of bacterial PNP [21]. Thus, cladribine is administered intravenously (IV). Following IV infusion, cladribine is rapidly internalized by cells via the hENT1, hENT2, hCNT2, and hCNT3 transporters and phosphorylated to the monophosphate form by dCK and deoxyguanosine kinase (dGK) [22-25], respectively. The relative role of dGK in mediating activation of cladribine is unclear in light of published data showing dGK is localized to the mitochondria whereas endogenous dCK is localized in the cytoplasm (exogenously overexpressed dCK results in nuclear localization) [26,27]. Thus, phosphorylation by dCK is thought to be a critical event that is responsible for both enriching cladribine inside the cell and preparing it for its cytotoxic actions [28]. The triphosphate form of cladribine is achieved after successive phosphorylation of the mono- and di-phosphate forms by the nucleoside monophosphate kinase and the nucleoside diphosphate kinase. Once the triphosphate is generated it serves as an effective substrate for DNA replication enzymes like DNA polymerases [29,30]. While incorporation of cladribine into DNA does not block chain extension *per se*, it is an inefficient substrate for extension and facilitates miss-incorporation of nucleotides [29]. Nevertheless, incorporation of cladribine into DNA leads to inhibition of DNA synthesis and, importantly, inhibition of DNA repair which in turn leads to formation of single strand breaks in DNA, poly(ADP-ribose) polymerase (PARP) activation and apoptosis via p53 dependent and independent pathways [31-33]. Interestingly, cladribine is toxic to both resting and actively proliferating cells (a feature shared with fludarabine) possibly in a p53 dependent manner [34]. In addition to its effects on DNA synthesis and repair, the tri-phosphate form of cladribine has also been shown to inhibit ribonucleotide reductase (RR) [30]. Inhibition of RR by cladribine results in depletion of deoxynucleoside triphosphate (dNTP) pools leading to further inhibition of DNA synthesis and inappropriate activation of endonucleases that promote formation of stand breaks [35]. Other mechanisms regarding the cytotoxic actions of cladribine have also been reported. Fabianowska-Majewska et al. reported that cladribine can inhibit deoxyadenosine deamination and phosphorylation suggesting a role in regulating deoxyadenosine metabolism [36]. Several groups have demonstrated that cladribine may directly damage the mitochondria, disrupt mitochondrial function and promote the release of AIF [37,38]. Despite the similarities in structure and mechanism to other nucleoside analogs, we are not aware of any successful clinical trials using cladribine as a radiosensitizer [39,40].

#### Clofarabine

Clofarabine (2-Chloro-9-(2-deoxy-2-fluoro-β-D-arabinofuranosyl)-adenine) is a deoxyadenosine analog used in the treatment of acute lymphoblastic leukemia (ALL) and acute myelogenous leukemia (AML) [41]. A number of favorable structural features of other nucleoside analogs were incorporated into clofarabine to help improve its pharmacokinetic profile and reduce toxicity, without altering pharmacodynamics [41,42]. For example, the addition of a fluoride atom to the 2’-position on the sugar group increases resistance to acidic conditions and bacterial PNP [43]. Also, reminiscent of modifications to cladribine and fludarabine, a halogen (chloride) at the 2-position of the adenine ring helps protect clofarabine from adenosine deaminase [43,44]. In addition, the presence of this chloride atom appears to enhance the catalytic efficiency of dCK for clofarabine [45].

After administration, clofarabine is transported into the cell by the concerted action of three types of nucleoside transporters, hENT1, hENT2, and hCNT2 [22,23]. Passive transport across the plasma membrane may also occur depending on the concentration of drug administered [22]. Upon entry into the cell, clofarabine undergoes rapid phosphorylation by dCK [41]. This is followed by rapid phosphorylation to the diphosphate form by purine nucleotide monophosphate kinase and to the triphosphate form by purine nucleotide diphosphate kinase [46]. The phosphorylation of clofarabine by dCK appears to occur with greater efficiency than fludarabine and cladribine [41,47,48]. Phosphorylation by dCK facilitates retention of clofarabine inside the cell thereby enriching intracellular drug concentrations. Indeed, the ABCG2 drug efflux pump can eject unphosphorylated clofarabine from the cell but not the monophosphate form [49]. As a result, the tissue distribution and relative expression levels of dCK in normal and cancer cells can influence both therapeutic efficacy and toxicity of clofarabine and other nucleoside analogs phosphorylated by dCK. Equally important is the fraction of active dCK present in normal cells as compared to cancerous cells. As discussed later in this review, while dCK is active in its native unphosphorylated state, its kinase activity is greatly enhanced when it is phosphorylated [50,51]. Once phosphorylated to the tri-phosphate form, clofarabine acts as a fraudulent nucleoside and is incorporated into DNA but serves as a poor substrate for subsequent addition of nucleosides onto the growing chain [52]. This, in turn, results in chain termination and strand breaks. The triphosphate form of clofarabine has also been shown to interfere with DNA polymerase-α, but not β or γ [52,53]. Finally, clofarabine-triphosphate is a potent inhibitor of ribonucleotide reductase (RR) that appears to result in an increase in clofarabine-triphosphate incorporation into DNA by depleting cellular concentrations of the normal, endogenous nucleotides [52,53]. Several studies suggest that clofarabine ultimately promotes induction of apoptosis through a combination of direct and indirect effects on the mitochondria [37,54]. As with the other radiosensitizing nucleoside analogs, *in vivo* activity against solid tumors have failed to reveal any objective responses in the absence of co-treatment with radiation [55]. However, the effectiveness of clofarabine for solid tumors has shown promise *in vitro* when used in tandem with radiation [56].

#### Nelarabine

Nelarabine is a prodrug of the guanosine analog, 9-β-D-arabinofuranosyl guanine (ara-G), that was granted accelerated approval by the FDA in 2005 for the treatment of T-cell ALL (T-ALL) and T-cell lymphoblastic lymphoma (T-LBL) [57]. Although ara-G was originally synthesized in the early 1960s its maturation into a viable clinical treatment modality was hindered because of its poor solubility [58]. However, in the 1970s work on human PNP deficiency rekindled interest in ara-G. Several key observations emerged from these studies: 1) human PNP deficiency can lead to depletion of T cells, 2) T cell cytotoxicity is associated with elevations in intracellular levels of dGTP (because dGTP is normally degraded by human PNP), and 3) B lymphocytes are largely unaffected, possibly as a result of differences in metabolism or cell cycle dependent accumulation of dGTP [59-63]. Based on these observations, it became apparent that T cells would be subject to killing by a guanine based analog, such as ara-G, which is not subject to degradation by human PNP [64]. Subsequent studies revealed that the cytotoxic actions of ara-G were principally directed towards T cells [64]. This was followed by the successful synthesis of nelarabine via addition of a methyl group to the N6 position of the guanine ring [58]. Upon administration, nelarabine is converted to ara-G by plasma localized ADA [58]. Ara-G readily enters cells via the hENT whereupon it is rapidly phosphorylated by either dCK or dGK, in a rate limiting manner, to the monophosphate form [65,66]. Phosphorylation to the di-phosphate and tri-phosphate forms may also be catalyzed by dGK [63,66]. The intracellular concentrations of ara-GTP appear to be highly dependent and related to kinase activity of dCK or dGK. An increase in dCK activity or dGK activity facilitates higher intracellular concentrations of ara-GTP which shifts the preference of ara-G from dGK to dCK [66]. Additionally, the presence of a β hydroxyl group at the 2’-position of the sugar moiety leads to high intracellular levels of ara-G by reducing its susceptibility to human PNP [64]. Recent studies have suggested that the ABCB1 transporter may play a role in development of resistance by pumping ara-G out of the cell although the prevalence of this mechanism is uncertain [67]. Nevertheless, once sufficient intracellular levels of ara-GTP are reached, incorporation of ara-GTP into DNA blocks chain extension leading to strand breaks and, ultimately, apoptosis [64,68]. Although there is currently no evidence that nelarabine or ara-G act as radiosensitizers, their reliance on dCK may make them subject to activation by the IR/ATM/dCK pathway thereby facilitating synergism as discussed below.

### Pyrimidine based analogs

#### Cytarabine

The deoxycytidine analog cytarabine (also known as ara-C or 1-β-arabinofuranosylcytosine) has been in clinical use for leukemias such as acute myelogenous leukemia (AML) since its synthesis in the late 1950s. Ara-C resembles endogenous deoxycytidine in all respects save for the position of the 2’-hydroxyl group on the sugar moiety which is in the arabinose configuration to distinguish it from cytidine. After administration, ara-C is primarily transported into cells via the human equilibrative transporter (hENT1) although this is thought to be concentration dependent [69]. At high concentrations, however, ara-C may enter the cell by passive diffusion [70]. Once inside the cell ara-C is phosphorylated in sequence to the triphosphate form by dCK and pyrimidine nucleotide kinases [71]. As with other nucleoside analogs, phosphorylation can serve as a means to retain ara-C in the cell however, it has been noted that phosphorylated ara-C can be effluxed from the cell by multi-drug resistance proteins (MRPs) 5 and 7 [72]. The importance of dCK mediated phosphorylation is supported by studies that show resistance to ara-C in cells lacking dCK [73,74]. Like other nucleoside analogs discussed thus far, it is the triphosphate form that is incorporated into DNA. Incorporation of ara-CTP into DNA occurs in competition with endogenous deoxycytidine tri-phosphate (dCTP) and once incorporated, the hydroxyl group on the ribose makes ara-CTP a poor substrate for chain extension. Ara-C appears to induce cell death via activation of the apoptotic program and inhibition of Bcl-2 expression re-sensitizes AML blasts to cell killing by Ara-C [75]. The mechanism of cell death may involve generation of reactive oxygen species [76]. As with ara-G, there is currently no evidence that ara-C can act as a radiosensitizer. However, because dCK is important for the activation of ara-C, there is a potential for chemosensitization by IR as discussed below.

#### Gemcitabine

The deoxycytidine analog, gemcitabine, is employed in the treatment of metastatic breast cancers, locally advanced or metastatic non-small cell lung cancers, pancreatic cancers, and relapsed ovarian cancers [77-84]. To address metabolic limitations of ara-C, gemcitabine was structurally modified through the addition of two fluorine atoms in lieu of a hydroxyl group on the 2’-position of the ribose. Similar to ara-C, gemcitabine is internalized into target cells via the human equilbrative nucleoside transporter 1 (hENT1) although other nucleoside transporters appear to also play an important role in uptake [24,85,86]. Also, like ara-C, gemcitabine is phosphorylated to the monophosphate form by dCK [87]. Gemcitabine-monophosphate is converted to the di-phosphate and tri-phosphate form in succession by pyrimidine nucleotide kinases [88,89]. Heinemann et al. demonstrated that, in contrast to ara-C tri-phosphate, gemcitabine tri-phosphate enters cells more rapidly, has a higher affinity for dCK and a slower elimination rate, leading to prolonged inhibition of DNA synthesis [87]. It has been documented that MRP 5 and 7 can pump gemcitabine out of the cell following internalization as a resistance mechanism [72]. Following phosphorylation to the triphosphate form, gemcitabine-triphosphate is incorporated into DNA leading to inhibition of DNA synthesis [90]. Interestingly, after the incorporation of gemcitabine-triphosphate into DNA a single, normal nucleotide is added to the 3’-hydroxyl of its ribose, shielding gemcitabine from DNA repair mechanisms including base excision repair [91]. The diphosphate form of gemcitabine is also a potent inhibitor of ribonucleotide reductase which leads to inhibition of DNA synthesis via depletion of deoxynucleotides [91]. As a consequence of this action, declining levels of dCTP de-inhibit dCK, increasing its activity favoring the generation of additional gemcitabine triphosphate [92]. Although the precise manner by which cell death is executed remains unclear, it is most likely mitochondrially mediated and caspase dependent [93,94].

## Mechanisms of synergism between nucleoside analogs and ionizing radiation

The clinical use of radiation in combination with chemotherapeutic agents gained significant momentum in the 1970s although many of the original studies date back to the early 1960s [95]. The underlying goal of these early efforts was a simple one, to synergistically increase tumor cell killing and improve patient outcomes [95]. The most studied means of synergism has been radiosensitization of cancer cells with nucleoside analogs. At the present time, gemcitabine, fludarabine, and clofarabine are employed clinically as radiosensitizers [96-100]. Observations from these and other studies have revealed mechanistic commonalities between these agents that contribute to radiosensitization including inhibition of DNA repair and modulation of nucleotide synthesis/availability. Ultimately, it is thought that these effects culminate in cell cycle redistribution/arrest and inhibition of DNA synthesis. While many questions remain unanswered concerning how these mechanisms work together to achieve radiosensitization this topic has been reviewed extensively elsewhere [100,101]. An alternative explanation of the synergism between radiation and nucleoside analogs, that remains underexplored, is IR-mediated chemosensitization. As discussed, not all nucleoside analogs act as radiosensitizers. Indeed, Nelarabine (ara-G), cytarabine (ara-C) and cladribine are not known to function as radiosensitizers despite having significant similarities in mechanism of action and metabolism to the radiosensitizing nucleoside analogs noted above. However, the ability to chemosensitize cells to these agents could represent an important strategy for synergism. Here we will briefly review the involvement of DNA repair inhibition in radiosensitization and contrast this mechanism with a recently identified pathway involving the ATM kinase and dCK that may lead to synergism through chemosensitization.

### Radiosensitization through inhibition of DNA repair

Inhibition of DNA repair is one method for nucleoside analog induced radiosensitization. Indeed, the inhibition of DNA repair pathways is a logical means by which these drugs could sensitize cancer cells to the DNA damaging actions of ionizing radiation. However, while DNA repair pathways remain an attractive target, there are few published examples of this type of inhibition by nucleoside analogs. The nature of the interaction between the DNA repair machinery and nucleoside analogs that leads to enhanced radiosensitization remains poorly described. For example, Wachters et al. used cells that were deficient in either XRCC2 or XRCC3 to show that gemcitabine interferes with homologous recombination (HR) repair pathways possibly by inhibiting Rad51 [102]. These same authors had previously reported that gemcitabine radiosensitization was not dependent on NHEJ and in fact radiosensitization was enhanced in the absence of an intact NHEJ system [103]. These results correlate well with cell cycle studies of gemcitabine that demonstrate maximal radiosensitization occurs in cells that have progressed into S phase when HR would be most active [104,105]. However, it is known that cells in S phase are more radioresistant compared to cells in other phases of the cell cycle. Also, there does not appear to be any significant increase in double strand break formation or repair with gemcitabine and radiation combination in tissue culture models [105,106]. This is in contrast with more recent studies demonstrating increased γ-H2A.X formation (a marker of double strand breaks) by gemcitabine and clofarabine in cells with siRNA silenced Neil1 [107]. Neil1 is a key glycosylase that initiates BER. Thus, the importance of gemcitabine mediated inhibition of HR in promoting radiosensitization and the explanation behind enhancement of radiosensitization in S phase remain to be fully elucidated. In the case of fludarabine, several publications have shown that it can inhibit BER [106,108]. A more recent study by Bulger et al. showed that the BER associated glycosylase UDG is upregulated in response to fludarabine in the leukemic cell line, HL60 [109]. Nevertheless, the consequences of fludarabines effects on BER in terms of radiosensitization remain unclear. Finally, a recent study by Stackhouse et al. examined the combination treatment with clofarabine and radiation where cell lines from several solid tumors were pre-treated with clofarabine for 1 hour followed by low dose IR treatment [56]. The most profound responses to this combination were seen in the head and neck cancer cell line SR475, the pancreatic cancer cell line PANC-1, and the colon cancer cell line HCT-116. The explanation as to how clofarabine radiosensitizes may relate to its ability to interfere with the DNA damage response and inhibit DNA repair [96]. Indeed, it has been reported that incorporation of clofarabine monophosphate into DNA may serve to inhibit DNA repair [41]. In general more studies are needed to validate the importance of DNA repair inhibition in mediating radiosensitization by nucleoside analogs. As a case in point, cladribine is known to inhibit DNA repair but it is a poor radiosensitizer.

### Role of IR-induced activation of deoxycytidine kinase in chemo- and radiosensitization

**Figure 1:**
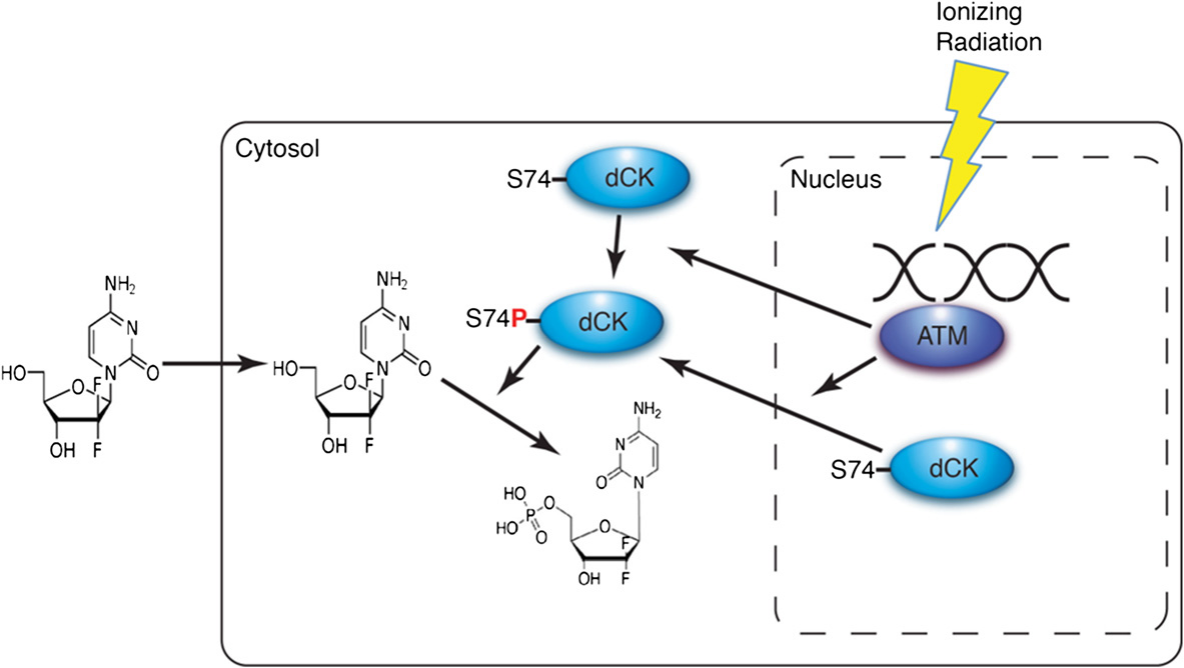
**Activation of dCK after radiation contributes to enhanced therapeutic effects of nucleoside analogs.** Treatment with ionizing radiation causes double strand breaks in DNA and activates the serine/threonine kinase Ataxia-telangiectasia mutated (ATM) which, in turn, phosphorylates deoxycytidine kinase, leading to a synergistic effect with nucleoside analogs. For example, gemcitabine enters the cell via the hENT1 transporter and is rapidly phosphorylated to the monophosphate form by active, serine 74 phosphorylated dCK prior to incorporation into DNA.

A number of publications have demonstrated that radiation alone can enhance the activity of dCK [110-112]. Interestingly, Csapo et al. show that increased dCK activity following low dose IR treatment is not a result of changes in dCK protein levels but rather due to post-translational modifications such as phosphorylation [110]. Given the critical role dCK plays in phosphorylating and activating agents such as gemcitabine, fludarabine, clofarabine, cladribine, nelarabine (ara-G), and cytarabine (ara-C) one would predict that cells with higher dCK activity (either intrinsically or via IR induction) would accumulate higher levels of active drug. This in turn would lead to enhanced cell cycle arrest, DNA damage by means of DNA repair inhibition or depletion of deoxynucleotide pools depending on the actions of the nucleoside analog in question. In support of this idea, Gregoire et al. went one step further by showing that increases in dCK activity directly correlate with radiosensitization exhibited by gemcitabine [97]. While these authors were able to demonstrate a tight correlation between the mRNA levels of dCK and radiosensitization the correlation between protein levels and radiosensitization was less robust again suggesting a role for post-transcriptional or post-translational modifications in dCK function and activity. The importance of post-translational modification of dCK was subsequently outlined in an eloquent series of studies by Bontemps and colleagues in which they demonstrated the role of phosphorylation in activating and stabilizing dCK kinase activity [50,113-115]. In these studies a number of candidate phosphorylation sites were identified including Thr-3, Ser-11, Ser-15, and most importantly, Ser-74 [50]. How dCK activation mediates the synergism between nucleoside analogs and radiation remained unclear until only recently. Our group has established that dCK can be phosphorylated by the DNA damage responsive kinase ATM on Ser-74, thereby directly linking radiation and dCK activation [51] (Figure 1). We further show that phosphorylated dCK can interact with and inhibit cyclin dependent kinase 1 (cdk1) which participates in governing the transition of cells from the G2 to M phase [116]. Thus increasing dCK activity via IR could potentiate synergism by creating a cellular environment favoring increased phosphorylation and activation of some, but not all nucleoside analogs [117]. Thus chemosensitization would occur as a result of the enhanced ability of nucleoside analogs to alter nucleotide synthesis and availability, cell cycle synchronization, and DNA repair processes. However, it is important to note that this synergism would not occur uniformly with all nucleoside analogs as evidenced by documented substrate preferences for S74 phosphorylated dCK or the S74E mutant [117,118]. Additionally, given the observation that maximal radiosensitization occurs when nucleoside analogs are administered prior to radiation activation of dCK by IR may represent a secondary event that propagates synergism rather than initiate it. Future studies are needed to fully understand the role that this signaling pathway plays in chemo- and radiosensitization and, ultimately, its clinical utility.

### Clinical application of deoxycytidine kinase as a biomarker and drug target

As noted above, many genes involved in DNA repair, DNA damage response, and activating nucleoside analogs have been determined to mediate the synergism between nucleoside analogs and radiation. Assessing how these genes and their resultant proteins are altered in cancer or in response to treatment offers the promise of identifying biomarkers to predict the potential susceptibility of individual patients to combination chemotherapy and radiotherapy. Additionally, gaining understanding of how these genes function to promote or impair chemosensitization or radiosensitization could yield insight into how to therapeutically enhance these processes using small molecule or gene therapy approaches. Focusing on deoxycytidine kinase, we will review the active efforts to identify variants of dCK that can drive the activation of nucleoside analogs and then follow this by a discussion of work towards establishing high-throughput screening methods for identification of therapeutic modulators of dCK.

Several groups have used pharmacogenomic approaches to identify genetic variants of dCK. Lamba et al. conducted an extensive examination of dCK single nucleotide polymorphisms (SNPs) in both European and African populations [119]. They identified a total of 64 genetic variants of which 3, I24V, A119G, and P122S, were nonsynonymous changes in the coding region. Further analysis of these variants revealed that I24V, A119G, and P122S exhibited significantly reduced ability to phosphorylate cladribine as compared to wild type dCK. The expression of these variants was examined clinically in patients with AML receiving ara-C either in short infusions or continuously. However, due to the low numbers of patients with these nonsynonymous polymorphisms their clinical significance remains unclear and further study is needed. A subsequent study by Kocabas et al. confirmed these results and analyzed the implications of these and other single amino acid changes (I24V, A119G, and P122S) on the structural conformation of dCK [120]. They note that these amino acid substitutions could alter the local flexibility and destabilize the conformation of dCK, however, the overall effect on dCK activity or as a phosphorylation target itself remain unclear. Li et al. identified an additional SNP in dCK (rs4308342) located in an intron that appears to be associated with altered sensitivity of lymphoblastoid cells from ethnically diverse populations to gemcitabine and ara-C [121]. These studies and others have helped pry open the door to discerning the relative contribution of individual amino acids in the function of dCK though it is evident that not all mutations in dCK have prognostic value as biomarkers [122]. Interestingly, none of the mutants identified in these screens had alterations in either the active site or phosphorylation sites of dCK such as serine-74. However, several publications have demonstrated that loss or attenuation of dCK activity can have profound implications on the activation of gemcitabine and ara-C. Indeed, independent studies by Saiki et al. and Ohmine et al. used matched pancreatic cell lines that were either sensitive or resistant to gemcitabine and then used gene expression and proteomic analysis approaches to define the role of dCK in gemcitabine resistance [123,124]. While it is becoming clear that dCK kinase activity is necessary for activation and efficacy of nucleoside analogs it is unclear if dCK phosphorylation site mutants are viable biomarkers or potential drug targets. However, phosphorylated dCK may be useful as a biomarker to gauge the functionality of dCK following radiotherapy but prior to administration of nucleoside analogs. Nevertheless, by gaining a more in depth understanding of how mutations in dCK alter its conformation and its ability to serve as a target for phosphorylation, it might be possible to screen or design small molecules to stimulate activation of dCK. However, key questions emerge such as: What types of dCK mutations might activate dCK? Can identification of dCK mutants with enhanced activity serve as a basis for small molecule drug design or gene therapy approaches?

To properly address these questions a high-throughput platform for identifying dCK mutants that have altered activity is needed. One such approach has been developed and tested by Rossolillo et al. who tested a retrovirus based system for generating screening libraries of gene mutants [125]. To validate their system they generated and identified mutant versions of dCK which, when over-expressed in cancer cells, alter susceptibility to gemcitabine. The most exciting mutant to emerge from this study is G12 a triple mutant that is altered at amino acids 171, 247, and 249 (E171K, E247K, and L249M). They demonstrate that although G12 phosphorylates gemcitabine as efficiently as wild type dCK, the G12 mutant exhibits significantly diminished ability to phosphorylate the endogenous dCK target, deoxycytidine as compared to wild-type dCK. Thus the G12 mutant is less likely to interfere with normal nucleotide synthesis catalyzed by dCK and instead is more directed towards gemcitabine activation. This suggests that the potential exists to modulate dCK to enhance its ability to phosphorylate nucleoside analog pro-drugs to active form. They also demonstrate that G12 has superior phosphorylation kinetics for gemcitabine compared to either the S74E mutant or the A100V, R104M, D133A triple mutant which also has altered substrate specificity [9,118,126]. They posit that because the E247 and L249 are located in the base sensing loop, which is thought to govern folding of dCK following binding of ATP or UTP, their mutation may explain the shift in substrate specificity seen with G12. Furthermore, the E171 residue located in alpha helix-7, which is involved in dCK dimer formation, may abrogate dCK dimer formation thus impairing its activity. Therefore this approach relies on generation of dCK mutants and validation of their activity prior to structural analysis.

An alternative approach that merits discussion uses insight gained from structural and functional studies of dCK to guide the design of dCK variants that can be expressed in cancer cells using gene therapy technology [127]. In this study, by Neschadim et al. dCK cDNA mutants were generated that exhibit altered activity and substrate specificity, and they were packaged them into lentiviral vectors for delivery to lymphoma or glioblastoma cells lines (Jurkat and MOLT-4 or U87mg, respectively). Mutation of dCK at arginine-104 and aspartic acid-133 have been previously demonstrated to alter the substrate specificity of dCK to include thymidine and deoxyuridine [126]. Still other studies have demonstrated that substitution of a glutamic acid residue in lieu of serine-74 leads to enhanced dCK activity by mimicking S74 phosphorylation [50,118,126]. For example, Hazra et al. sought to ascertain if expressing dCK double (R104M and D133A) or a triple (R104M, D133A, and S74E) mutants in cancer cells would increase the sensitivity of dCK to non-natural substrates like pro-drugs bromovinyl-deoxyuridine (BVdU) or L-thymidine (LdT) [128]. The cells transduced with triple mutants were most sensitive to cell death in response to treatment with both BVdU and LdT. The glioblastoma cell line U87mg was most sensitive followed by both lymphoma cell lines. These studies, therefore, offer proof of concept that dCK can serve as a potential biomarker or target for small molecule development.

## Conclusions

In summary, a number of prominent nucleoside analogs have been shown to have a synergistic effect when used in combination with radiation. The underlying mechanisms behind this synergism remain poorly understood but may result from inhibition of DNA repair machinery, inhibition of DNA synthesis, cell cycle redistribution, or activation of nucleoside kinases such as dCK. Indeed, many of the currently used nucleoside analogs that have exhibited synergistic activity with radiotherapy are activated by dCK. It is well recognized that hematological malignancies, including many leukemias and lymphomas, express higher than normal levels of dCK and that this makes them more “sensitive” to nucleoside analog induced cell death. However, solid tumors do not exhibit a clear dCK expression pattern and in many cases they have low dCK expression levels. Thus, by leveraging recent developments in our understanding of dCK function and activation it may be possible to develop pharmacologic or genetic therapeutic approaches to increase the susceptibility of these tumors to radiation and antimetabolite combination therapy. Additionally, new insight on the function of dCK and mechanism of activation has applicability to nucleoside analogs in the pipeline currently such as thiarabine and sapacitabine.

## Competing interests

Southern Research Institute holds the patent on clofarabine and has received royalties from its sale. William B. Parker has financially benefitted from these payments. Bo Xu has a US Patent Application (US2008/0262003A1) related to clofarabine.

## Authors’ contributions

BX and MWL organized the structure of the manuscript. ML, WBP and BX wrote the manuscript. All authors read and approved the final manuscript.
